# Interdisciplinary complication management of dislodged lumbar interbody spacer in pulmonary artery

**DOI:** 10.1259/bjrcr.20170047

**Published:** 2017-11-17

**Authors:** Andreas Kunz, Thorsten Klink, Stefan Köhler, Richard Kellersmann, Christian Markus, Thorsten Bley, Ralph Kickuth

**Affiliations:** ^1^Department of Diagnostic and Interventional Radiology, University Hospital Wuerzburg, Wuerzburg, Germany; ^2^Department of Neurosurgery, University Hospital Wuerzburg, Wuerzburg, Germany; ^3^Department of General, Visceral, Vascular and Pediatric Surgery, University Hospital Wuerzburg, Wuerzburg, Germany; ^4^Department of Anesthesia and Critical Care, University Hospital Wuerzburg, Wuerzburg, Germany

## Abstract

We report the case of an intraoperatively dislodged transforaminal lumbar interbody fusion spacer with creation of a traumatic arteriovenous fistula and device migration to the pulmonary artery. Successful minimally invasive angiographic retrieval of the spacer is discussed with special reference to angiographic and surgical treatment strategies and pitfalls.

## Introduction

Minimally invasive transforaminal lumbar interbody fusion (TLIF) has become a widely employed technique for the surgical treatment of degenerative spondylolisthesis, promising long-term symptomatic pain relief. Apart from the general medical risks associated with surgery, common specific complications are known to include haemorrhage and neurological symptoms, as well as spacer misplacement and dislodgement. Whether conventional open surgery or newer minimally invasive techniques provide superior outcomes remains inconclusive to date.^1^

### The case

**Figure 1.**
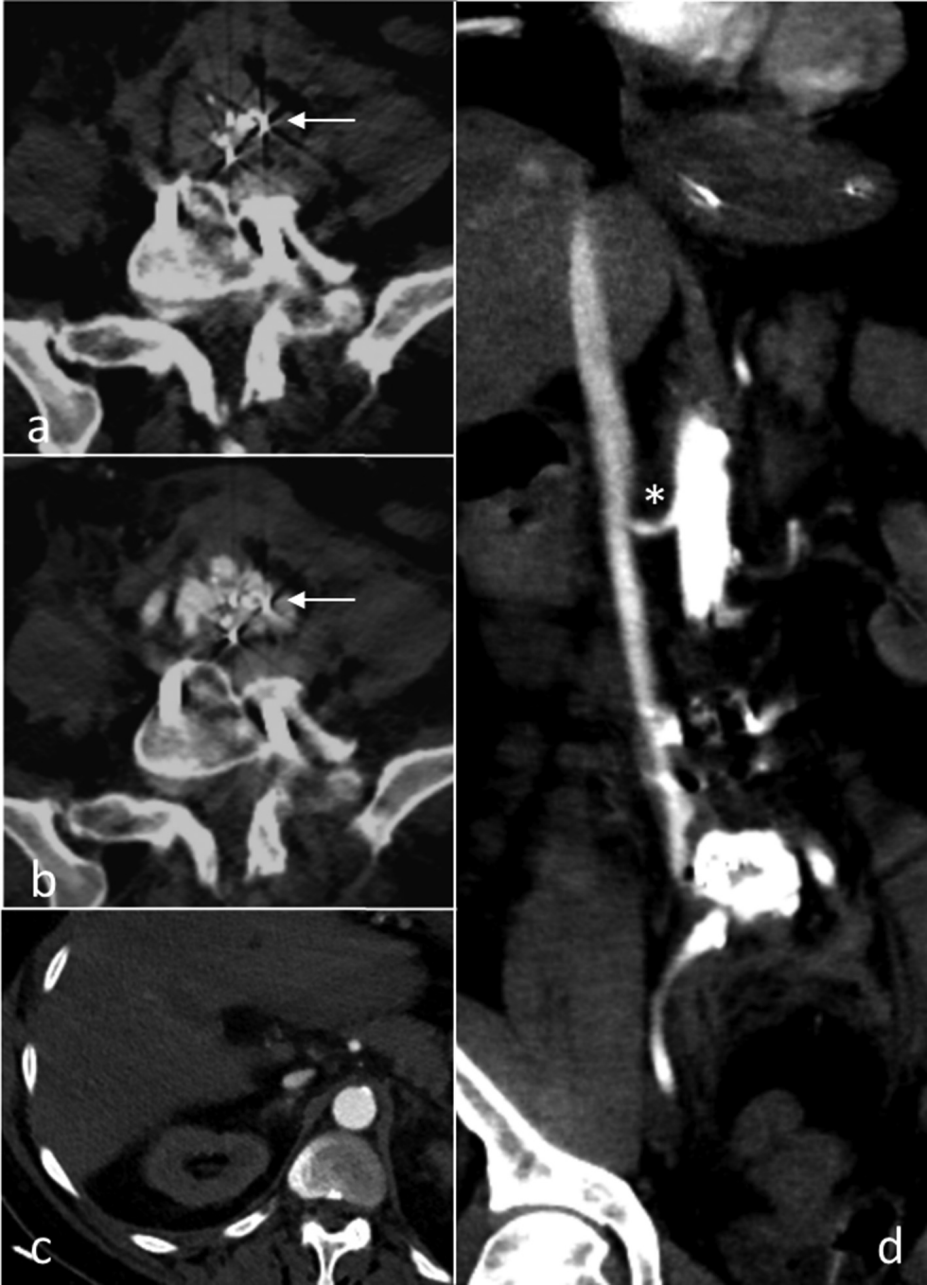
CT scan immediately after conclusion of surgery in prone position. Non-contrast-enhanced (a) and contrast-enhanced (b) scans show the dislodged transforaminal lumbar interbody fusion spacer in the prevertebral space (arrows). Significant vascular trauma has led to the formation of a pseudoaneurysm with incorporation of the spacer. Early arterial contrast phase documents extensive contrast within the inferior vena cava (c, d) up to the diaphragm indicating an arteriovenous fistula. Please note that the right renal artery (star) is merely located in close proximity to the inferior vena cava (d) without further vessel anomaly.

We report the case of a 58-year-old female with intractable back and leg pain and paresis of the left lower limb due to instability of the adjacent segment L4/5 after spondylodesis L2-4 one year earlier. Conventional dorsal instrumentation and TLIF L4/5 was performed. During nucleotomy of L4/5 the patient presented a brief, self-limiting episode of haemodynamic instability. The underlying reasons remained unclear at that time. Later, the TLIF spacer dislodged into the prevertebral space, although placement thereof was performed without force and no resistance was perceived. Recovery of the dislodged spacer was impossible for the performing surgeons. As the patient immediately became haemodynamically unstable, surgery was aborted and a contrast-enhanced CT scan was performed with the patient remaining in prone position. The dislodgement of the spacer had caused a traumatic pseudoaneurysm between the right common iliac artery and inferior vena cava and incorporation of the spacer within. The scan revealed only minor retroperitoneal haematoma [Figure 1].

As the patient had become increasingly haemodynamically stable, interdisciplinary consensus was reached to closely monitor the patient without immediate surgical retrieval of the spacer via laparotomy.

**Figure 2.**
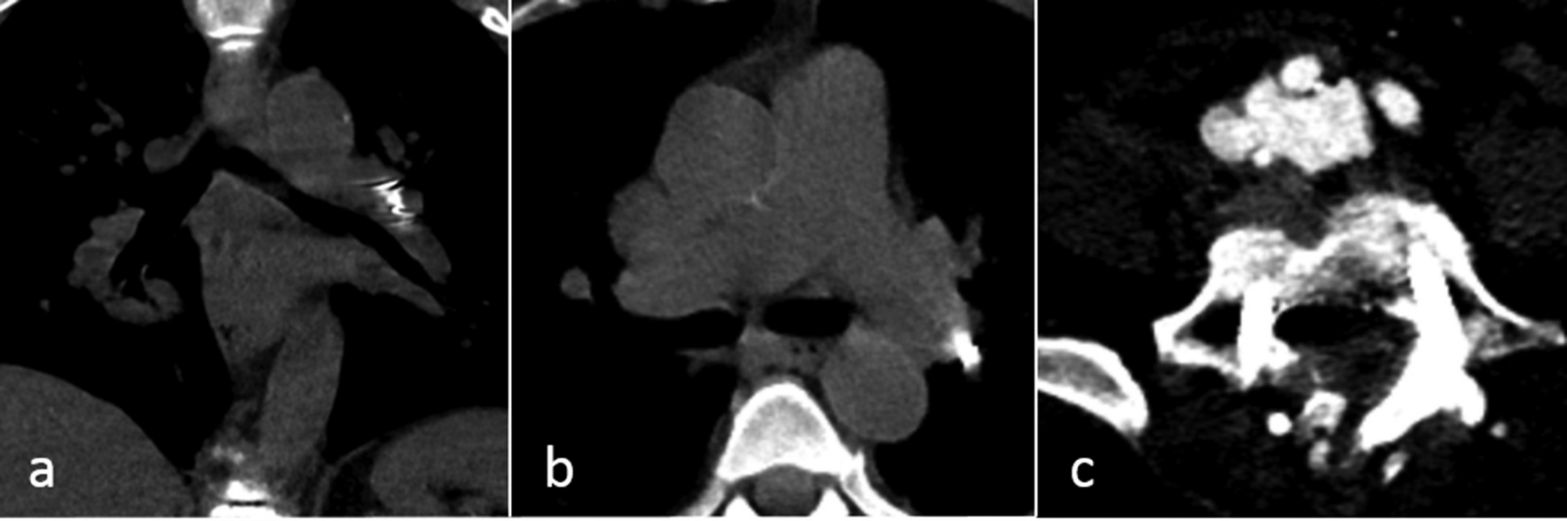
CT scans on the 2nd postoperative day. (a, b) show coronal and transversal non-contrast-enhanced CT images of the chest. The transforaminal lumbar interbody fusion spacer (arrows) has embolized to the left pulmonary artery, partly extending into the lower lobe artery. Contrast-enhanced image (c) of the prior transforaminal lumbar interbody fusion location proves its embolization. The arteriovenous fistula originating from the right common iliac artery is now more clearly identifiable.

A follow-up CT scan on the second postoperative day revealed that the spacer had not only been incorporated into the venous system but had embolized in the left pulmonary artery, partly extending into the lower lobe artery [Figure 2]. Interdisciplinary consensus was reached between interventional radiology, thoracic surgery, vascular surgery and anaesthesiology departments to attempt an intravascular retrieval of the spacer via inguinal venotomy. The rationale for this proceeding was that abdominal vessel trauma was feared to prohibit the use of necessary life-support machines during open thoracotomy with an aimed activated clotting time of 500 s. Treatment strategy was discussed with the patient at length, who concurred.

**Figure 3.**
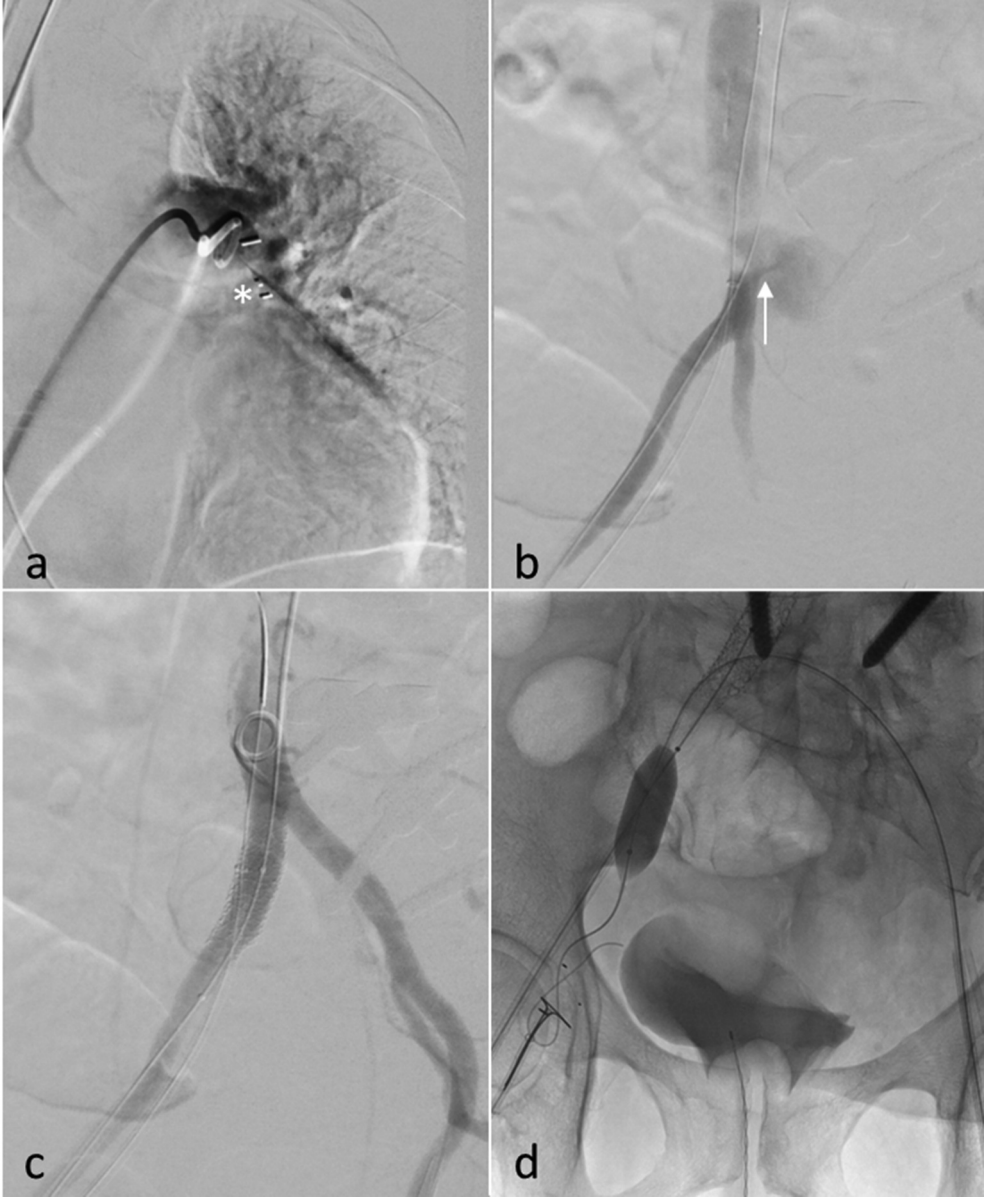
Angiographic images of the transforaminal lumbar interbody fusion (TLIF) retrieval procedure: (a) The wedged TLIF spacer (arrow) is embolized to the left pulmonary artery. (b) Arteriovenous fistula (star) originating from the right common iliac artery. (c) Occlusion of the arterial shunt feeder by application of overlapping covered stents due to residual arteriovenous fistula after initial stenting (10/20 mm Advanta stent and 11/38 mm Advanta stent). The right internal iliac artery is consecutively occluded. (d) Retrieval of the TLIF spacer and retraction thereof into the right femoral vein utilizing an Amplatz GooseNeck® Snare Kit. Re-migration of the spacer during venotomy was prevented by introducing a venous balloon catheter via the contralateral groin in order to temporarily occlude the right iliac vein.

The primary angiographic procedure was performed via the right iliac vein utilizing a 7F sheath. The TLIF spacer, which continued to be located within the left pulmonary artery [Figure 3a], was grasped by means of a snare catheter (Amplatz GooseNeck^®^ Snare Kit, Medtronic Minimally Invasive Therapies Group, Mineapolis, Minnesota, USA), mobilized and retrieved through the inferior vena cava. However, the snare kit became lodged when passing the pelvic region and the patient instantly became severely haemodynamically unstable. The combination of pushing the snare back into the inferior vena cava and i.v. application of high-dose catecholamines alleviated the condition promptly, however. Secondary vessel trauma to the venous system and the pulmonary artery was ruled out by direct angiography.

As a conceivable cause for the unanticipated destabilization, we hypothesized a pre-existing pathologically high cardiac preload due to the AV fistula and resulting shunt mechanism between right iliac artery and inferior vena cava. Plausibly, the patient would have accommodated to this condition since the first operation. At the time, the lodged snare kit would have instantaneously impeded blood flow through the shunt, leading to destabilization and drop in blood pressure. To test the discussed pathophysiological alterations resulting in critical hypotension, the previously diagnosed traumatic AV fistula originating from the right common iliac artery [Figure 3b] was temporarily occluded with an inflated balloon. Almost immediately, blood pressure levels increased significantly, spiking to as much as 300 mm Hg systolic value. Henceforth, catecholamine therapy was discontinued.

To permanently normalize blood flow within the inferior vena cava and the pre-existing pathologically high levels of cardiac preload, as well as to facilitate TLIF spacer retrieval, a covered stent was placed over the arterial fistula (10/20 mm Advanta® stent). Consecutive angiographic visualization revealed a persisting AV shunt, so another covered stent was placed in overlapping technique (11/38 mm Advanta® stent, Maquet, Rastatt, Germany), resulting in occlusion of the arterial side of the shunt and covering of the right internal iliac artery [Figure 3c].

Re-migration of the spacer during venotomy was prevented by introducing a venous balloon catheter via the contralateral groin in order to occlude the right iliac vein [Figure 3d]. Consecutive venotomy and spacer retrieval were performed by a vascular surgeon. Repeated direct angiographies during the intervention showed no lung artery embolism or secondary vessel trauma. Postinterventional CT scans confirmed the interventional success and denied secondary complications. ICU monitoring was upheld for a total of 7 days after the initial operation. 8 days thereafter the patient was released from hospital treatment in good general condition, with significant pain relief and improvement of neurological deficits.

Ethics commission approval for the above described treatment was waived according to our institution’s guidelines.

## Discussion

To our knowledge, the described complications regarding intravascular device embolization after ventral TLIF spacer dislodgement are novel to literature. Prevertebral vessel trauma during nucleotomy is a widely known and feared complication of the performed procedure. Conceivably, spacer dislodgement was facilitated by pre-existing damage of the ventral longitudinal ligament caused by lumbar segmental instability. During transition from the operating room to the radiology department for damage assessment the patient remained in prone position as repositioning has been described to further jeopardize the haemodynamic state after vessel trauma has occurred.^2 ^

From a neurosurgical perspective, the complication encountered stresses the importance of proceeding with excessive caution during preparation and implantation of TLIF devices. This is especially the case if any detail may hint at possible vessel trauma even in the case of persisting or spontaneously regained haemodynamic stability. Looking back, the brief episode of instability during nucleotomy may have been indicative of exactly that.

Treatment planning was discussed in an interdisciplinary setting. In particular, the risk of intraprocedural complications was deemed significant, especially the risk of vessel trauma in both thoracic and abdominal locations, as well as intracardiac damage (*i.e.* damage to both the pulmonary and tricuspid valves), requiring detailed contingency plans.

However, the above-mentioned risks did not outweigh the reservations regarding open thoracotomy, as necessary employment of life-support machines would have required significant anticoagulation in the setting of significant vascular trauma with the risk of uncontained haemorrhage. In retrospect, angiographic occlusion of the iliac shunt and retrieval of the spacer proved to be an elegant, minimally invasive treatment option minimizing secondary trauma to the patient. The preformed pseudoaneurysmatic cavern was fully resorbed, as diagnosed in a second postinterventional CT scan, so that no further treatment is scheduled at this time.

## Learning points

1. Transforaminal lumbar interbody fusion (TLIF) spacer is a viable surgical procedure for the treatment of degenerative spondylolisthesis promising long-term symptomatic relief.
2. Accompanying risks during surgery include spacer dislodgement into the prevertebral space, especially in the case of pre-existing damage to the ventral longitudinal ligament.
3. Unusual, dramatic migration and associated vascular injury with consecutive spacer incorporation into the venous system are extremely rare; however, such complications require interdisciplinary efforts for their resolution.

## Consent

Written informed consent for the case to be published (including images, case history and data) was obtained from the patient(s) for publication of this case report, including accompanying images.
